# Editors Should Declare Conflicts of Interest

**DOI:** 10.1007/s11673-019-09908-2

**Published:** 2019-04-23

**Authors:** Jaime A. Teixeira da Silva, Judit Dobránszki, Radha Holla Bhar, Charles T. Mehlman

**Affiliations:** 1 P.O. Box 7, Miki-cho post office, Ikenobe 3011-2, Kagawa-ken, 761-0799 Japan; 2 Research Institute of Nyíregyháza, IAREF, University of Debrecen, P.O. Box 12, Nyíregyháza, H-4400 Hungary; 3 Alliance Against Conflict of Interest, BP 33, Pitampura, Delhi, 110 034 India; 4 Division of Pediatric Orthopaedic Surgery, Cincinnati Children’s Hospital Medical Center, Cincinnati, OH USA

**Keywords:** Accountability, Editorial responsibility, Peer review, Quality control, Transparency

## Abstract

Editors have increasing pressure as scholarly publishing tries to shore up trust and reassure academics and the public that traditional peer review is robust, fail-safe, and corrective. Hidden conflicts of interest (COIs) may skew the fairness of the publishing process because they could allow the status of personal or professional relationships to positively influence the outcome of peer review or reduce the processing period of this process. Not all authors have such privileged relationships. In academic journals, editors usually have very specialized skills and are selected as agents of trust, entrusted with the responsibility of serving as quality control gate-keepers during peer review. In many cases, editors form extensive networks, either with other professionals, industry, academic bodies, journals, or publishers. Such networks and relationships may influence their decisions or even their subjectivity towards a set of submitting authors, paper, or institute, ultimately influencing the peer review process. These positions and relationships are not simply aspects of a curriculum, they are potential COIs. Thus, on the editorial board of all academic journals, editors should carry a COI statement that reflects their past history, as well as actual relationships and positions that they have, as these may influence their editorial functions.

Not knowing what constitutes best practice is incompetence. Knowing what best practice is, but not knowing how to achieve it, may be inexperience. Knowingly not following best practices, when one knows how to achieve it, is unethical. (Smith 2002, 205)

## The Central Role of Editors in the Publishing Process and Their Evolving Responsibilities

The structure of an editorial board of a scholarly, academic journal usually consists of a leading person, an editor in chief (EiC), senior or managing editors, associate editors, and advisory editors. There may also be linguistic editors, statistic editors, and even image or technical editors. All these editors carry responsibilities, for which they are given credit, and thus recognition, as academic acclaim, for being editors (Teixeira da Silva 2013). In this paper, in order to simplify the discussion, the term “editors” will be used broadly to describe any editorial position, independent of rank, although we recognize that there is a chain of command that also reflects a level of responsibility, with the EiC usually being ultimately responsible for all editorial decisions and for what eventually gets published in their journal.

In the past few years, a series of scandals as a result of failed peer review (Teixeira da Silva and Dobránszki 2015), fake peer reviews, false authorship, or other faked aspects of the publishing process (Teixeira da Silva 2017a), as well as a string of retractions—which generally reflect failure (Teixeira da Silva 2016b)—have occurred in academic publishing. To some degree, there is both a crisis of trust in science, not only within academia but also by the public since public (tax payer) funding supports various scientific structures around the world.

According to a white paper published by the Council for Science Editors (CSE), editors, especially of scientific journals, have responsibilities towards authors, peer reviewers, the journal itself, advancement of science, and the general public (Council of Science Editors 2019). The CSE updated that white paper in May of 2018 to add greater details about editors’ responsibilities when dealing with authors’ and their own conflicts of interest (COIs). The *Croatian Medical Journal* (CMJ 2009) places the entire responsibility for the content of a journal on editors’ shoulders. The World Association of Medical Editors (WAME) explains that a COI exists when:

… there is a divergence between an individual’s private interests (competing interests) and his or her responsibilities to scientific and publishing activities such that a reasonable observer might wonder if the individual’s behavior or judgment was motivated by considerations of his or her competing interests. COI in medical publishing affects everyone with a stake in research integrity including journals, research/academic institutions, funding agencies, the popular media, and the public. (World Association of Medical Editors 2009, ¶1)

In a stage of science that appears to be in a state of mistrust (Funk 2017) related to the integrity of the published literature, under-powered analyses, publication selection biases, and fraud (Wicherts 2017), including image manipulation (Teixeira da Silva 2016a), image editors carry new responsibilities. For example, the *Journal of Cell Biology* discovered that “about 1% of accepted papers had manipulated images that affected their conclusions; another 25% had some sort of manipulation that violated guidelines” (Couzin-Frankel 2016, ¶3 under “A change of plans”). Perceiving this crisis, and in an attempt to deal with public complaints about possible image manipulation in papers published by the *Journal of Biological Chemistry*, the American Society for Biochemistry and Molecular Biology made Kaoru Sakabe the data integrity manager, formerly the manager of publication issues; then, in April of 2017, it placed a call for three “Technical Image Editors.”^1^ In a bid to shore up trust and create a literature that is based on the quality and integrity of the published literature, *Molecular and Cellular Biology* (MCB) fortified their editorial functions and responsibilities, while abandoning the journal impact factor, by committing to post-publication peer review (PPPR) in which possible errors in the published literature would be freshly examined in the light of complaints made by academics or the public (Kullas and Davis 2017). And, at a risk to their own reputation, MCB concluded, based on inappropriate figure manipulations in 6.1 per cent of papers within their own journal, that “as many as 35,000 papers in the literature are candidates for retraction due to inappropriate image duplication” (Bik et al. 2018, p. 1).

Several associations of science editors and scientific journals have updated their COI policies to deal with the challenges listed above. These include disclosing publicly the actual or perceived COIs of the EiC or of other members of the editorial board, which may include financial relationships (including consultancy, honoraria, affiliations, past employment or association, and stock ownership), personal relationships, and non-financial COIs. A non-financial COI can arise when the editors are in situations such as having an unpaid membership in a board, government, or committee, if they have earlier co-authored papers with the author of the paper submitted for review, or if they have worked in institutions where the author works. It can also stem from desires to return favours or seek status and fame (Wiersma et al. 2018). A 2008 *PLoS Medicine* editorial noted that several of the COI cases brought before the Committee on Publication Ethics (COPE) involved non-financial COIs (*PLoS Medicine*^2008^). Non-financial COIs can be personal, political, academic, ideological, or religious (*PLOS Medicine*^2008^; Gallo et al. 2016) and are more difficult to define than financial COIs. A comprehensive definition which includes all forms of COIs in academic publishing is the one used by the Public Library of Science (PLoS) for its journals:

A competing interest is anything that interferes with, *or could reasonably be perceived as interfering with*, the full and objective presentation, peer review, editorial decision-making, or publication of research or non-research articles submitted to PLOS. Competing interests can be financial or non-financial, professional, or personal. Competing interests can arise in relationship to an institution, organization, or another person. (PLOS One 2019, ¶1, emphasis ours)

The difficulties in identifying and managing non-financial COIs have led to controversy regarding whether they should be managed at all. Wiersma et al. (2018) point out that non-financial COIs such as the potential for attaining status are often even more powerful in influencing research than financial COIs. On the other hand, critics of this increased attention to non-financial COIs claim that focus on non-financial COIs can detract attention from obvious financial COIs (Bero and Grundy 2016). In the context of research, Bero and Grundy believe that intellectual interests of the researcher cannot be separated from the researcher, and that these are essential for rigorous research and healthy scientific debate and should not therefore be perceived as COIs. However, we believe that this does not hold true for editors, as such conflicts, or interests, can bias or influence the publication of research. Our view is supported by the notion that intellectual stand-points can also reflect COIs (Lenzer 2016). It is not helpful that the term COIs be used simply, but erroneously, to describe influencing factors in the publishing process (Amigo and Pascual-García 2017).

An editor has responsibility towards authors, peer reviewers, the journal, and the public at large or the scientific community the journal is aimed at (Teixeira da Silva and Dobránszki 2018). In many cases of COIs in editors, the judgement of the editor can affect the future of the journal itself and, especially in the case of biomedical journals, it can affect peoples’ lives. In an opinion piece in *JAMA*, McCoy and Emanuel (2017, 1721) stated that

… [t]he notion of a potential conflict of interest reflects the mistaken view that a COI exists only when bias or harm occurs. This way of reasoning confuses a real situation marked by a potential for bias with a potential situation.

Gottlieb and Bressler (2017), in the same edition of *JAMA*, asked “who watches the watchers?”—noting that editors shape biomedical literature by deciding which articles, commentaries, opinion pieces, reviews, and letters to the editor will be published and when, and that such literature can have an influence on healthcare. Hence the potential of biased decision-making by an editor or his or her COIs should be a matter of concern (Gottlieb and Bressler 2017). Rumsey (1999) noted that editors and reviewers have a duty to maintain the integrity of the publishing process and thus must contend with past biases in literature caused by incomplete reporting, favouritism, failure to consider other explanations of scientific results, failure to report negative or statistically nonsignificant studies, publication of preliminary results that are refuted in later studies, and situations unique to the project funding process. Perceptions of the existence of bias such as a delay in publishing to meet personal needs may, in the long run, affect the journal itself (Rumsey 1999).

Sarigöl et al. (2017) described how prior relationships between an editor and an author can favour faster publication times, suggesting that hidden COIs exist in such relationships. Since publishing is both a stressful and time-consuming process, such unfair treatment in favour of one party (and thus against other parties who do not receive such preferential treatment) can corrupt the publishing process because they are intrinsically biased and unfair. Consequently, given the central importance of editors in the publishing process, as well as in paper selection and decision-making, editors should be expected to display not only their curriculum vitae in its entirety without any selective bias or skewing of the facts (Teixeira da Silva and Tsigaris 2018) but also their COIs.

Given that almost everyone has competing interests and biases and the fact that they often overlap with personal and non-financial COIs makes their management difficult. One method is full disclosure, where authors, reviewers, and editors acknowledge having non-financial interests which might influence their reporting or review, or where there is a possibility that the publication of the paper will either positively or negatively impact their interest (*PLoS Medicine* Editors 2008). However, disclosure of non-financial COIs is not required by many journals, while financial COIs are, as noted by both Kesselheim et al. (2012) and Bosch et al. (2013).

While Bero and Grundy (2016) believe that recusal can be exclusionary by giving higher priority to interests opposing that of a researcher, so guidelines set by several journals suggest recusal as a means of managing both financial and non-financial COIs (*Journal of Medical Imaging and Radiation Sciences*^2013^). The CSE white paper specifies that a COI exists where editors make decisions on research manuscripts from their department or research collaborators or competitors or address an issue of particular interest to the editor. It suggests that such manuscripts should be delegated to other editors. Elsevier’s *Journal of Accounting and Economics* hands over decision-making to co-editors who are least conflicted if individual editors are found to have a COI, while manuscripts are also assigned to referees to reduce the potential for COIs (JAE 2017). This appears to be a general rule for all Elsevier journals (table 1). The *Croatian Medical Journal* has evolved a system of independent editorial review, often handing over the review process to an outside editor so that the risk of bias can be prevented (CMJ 2009). COPE only requires that journals provide the full names and affiliations of the journal’s editors on the journal’s web site as well as contact information for the editorial office (COPE 2011).

**Table 1:** Conflict of interest (COI) policy for editors, authors and reviewers as indicated by different publishers

Publisher	COI policy, sheet or statement/declaration	Pertain to editors (E), authors (A) or reviewers (R)
Bentham ^[1]^	“Author’s responsibilities: … Any potential conflict of interest must be clearly acknowledged. …” (Bentham 2017a, 2018)	A
	No exact rule for editors for stating their own COI. “Financial contributions to the work being reported should be clearly acknowledged, as should any potential conflict of interest.” (Bentham 2017b); “The editor should evaluate manuscripts objectively based on their academic merit free of any commercial or self-interests” (Bentham 2018).	R
	Clear statement for reviewers: “*Bentham Science* respects requests not to have the manuscripts peer-reviewed by those experts who may have a competing interest with the author(s) of a submitted manuscript. It is not possible for Editors to be aware of all competing interests; we therefore expect that reviewers would inform the Editor-in-Chief/Handling Editor if they notice any potential competing interest during the course of review of a manuscript. Moreover, the reviewers are expected to inform the Editors or editorial office of the journal if they have a conflict of interest in carrying out a review of a manuscript submitted by any author/contributor of the manuscript.” (Bentham 2017c, 2018); “Notifying the journal editor about any financial or personal conflict of interest and declining to review the manuscript when a possibility of such a conflict exists.” (Bentham 2018).	
De Gruyter ^[2]^	“In order to encourage transparency without impeding publication, all authors, referees and editors must declare any association that poses a conflict of interest in connection with the manuscript. There should be no contractual relations or proprietary considerations that would affect the publication of information contained in a submitted manuscript. A competing interest for a scholarly journal is anything that interferes with, or could reasonably be perceived as interfering with, the full and objective presentation, review, or publication of research findings, or of articles that comment on or review research findings. Potential conflicts of interest exist when an author, editor or reviewer has financial, personal or professional interests in a publication that might influence their scientific judgment.	E, A, R
	Examples of such conflicts include, but are not limited to:	
	• Financial conflicts: stock ownership; patents; paid employment or consultancy; board membership; research grants; travel grants and honoraria for speaking or participation at meetings; gifts	
	• Personal conflicts: relationship with editors, editorial board members, or with possible reviewers who have had recent or ongoing collaborations with the authors, have commented on drafts of the manuscript, are in direct competition, have a history of dispute with the authors	
	• Professional conflicts: public associations with institutions or corporations whose products or services are related to the subject matter of the article; membership of a government advisory council/committee; relationship with organizations and funding bodies	
	Authors should declare whether they have any conflicts of interests that could have influenced the reporting of the experimental data or conclusions in their paper. Such a statement should list all potential interests or, if appropriate, should clearly state that there are none. The editors may decide not to publish papers when we believe the competing interests are such that they may have compromised the work or the analyses or interpretations presented. Upon submission of a manuscript, authors may suggest to exclude any specific editors or reviewers from the peer review of their article. It is the responsibility of authors to disclose in the Acknowledgments section any funding sources for the project or other relationships that are relevant. Authors are suggested to fill in the Conflicts of Interest Form and send the electronic version to the Journal Editor.	
	Editors should consider whether any of the above competing interests are relevant to them and the manuscript under consideration. Editor who believes that the conflict will preclude an impaired judgment should disclose to the Editor the nature of the conflict and decline to handle the paper.	
	Reviewers should consider whether any of the above applies to them and declare any such competing interests. If they feel they cannot review a paper because of any competing interest, they should tell us. They should also declare any association with the authors of a paper.” For EICs: “Unpublished materials disclosed in a submitted manuscript must not be used in an Editor’s own research without the explicit written consent of the author(s)” For Peer Reviewers: “Privileged information or ideas obtained through peer review must be kept confidential and not used for personal advantage. Reviewers should not consider evaluating manuscripts in which they have conflicts of interest resulting from competitive, collaborative, or other relationships or connections with any of the authors, companies, or institutions connected to the submission.” For Authors: “All authors should disclose in their manuscript any financial or other substantive conflict of interest that might be construed to influence the results or their interpretation in the manuscript. All sources of financial support for the project should be disclosed.” (De Gruyter 2018)	
Elsevier ^[3]^	“Journal submissions are assigned to editors in an effort to minimize potential conflicts of interest. The following relationships between editors and authors are considered conflicts and are avoided: Current colleagues, recent colleagues, recent co-authors, and doctoral students for which editor served as committee chair. After papers are assigned, individual editors are required to inform the managing editor of any conflicts not included in the list above. In the event that none of the editors satisfy all of the conflict screens, co-editors who are least conflicted will be assigned to the manuscript. In addition, co-editors who are least conflicted are assigned for all paper submissions by sitting editors. Journal submissions are also assigned to referees to minimize conflicts of interest. After papers are assigned, referees are asked to inform the editor of any conflicts that may exist.”(Elsevier 2017a) + FACTSHEET Conflict of Interest (Elsevier 2017b). “Any potential editorial conflicts of interest should be declared to the publisher in writing prior to the appointment of the editor, and then updated if and when new conflicts arise. The publisher may publish such declarations in the journal. The editor must not be involved in decisions about papers which s/he has written him/herself or have been written by family members or colleagues or which relate to products or services in which the editor has an interest. Further, any such submission must be subject to all of the journal’s usual procedures, peer review must be handled independently of the relevant author/editor and their research groups, and there must be a clear statement to this effect on any such paper that is published [10]. The editor shall apply Elsevier’s policy relating to the disclosure of potential conflicts of interest by authors and reviewers, e.g. the International Committee of Medical Journal Editors (ICMJE) guidelines [1].” (Elsevier 2017c)	E, A, R
Emerald ^[4]^	“All conflicts of interest should be declared by the author, editor or reviewer.	E, A, R
	Conflicts of interest include:	
	• A financial or personal interest in the outcomes of the research;	
	• Undisclosed financial support for the research by an interested third party;	
	• A financial or personal interest in the suppression of the research;	
	A note to highlight the background to financial support for the research from third parties or any other possible conflict of interest must be added to the paper prior to review. If a conflict of interest is suspected, then this should be reported to the editor or Emerald. A concern regarding an editor should be raised with the journal publisher or book commissioning editor at Emerald. Emerald will follow the flowcharts presented by COPE in cases of a suspected conflict of interest.”	
Hindawi ^[5]^	Managing Conflict of Interest	E, A, R
	“Authors Conflicts for authors are most often associated with the risk of bias in a manuscript. As an author, if you have any interest or association that could be seen to have influenced your decision-making process, you should ensure that it is declared at the time of submission. …Whether or not you believe a conflict of interest exists, you will be asked to include a statement in your manuscript. If you believe no conflicts exist, you will be asked to confirm this in writing. Editors As a member of a journal’s Editorial Board, you need to be very aware of the risk of conflicts when handling a manuscript. Firstly, you should assess your own potential conflicts. If you have recently coauthored with the author of the manuscript, you could be perceived to be influenced by your relationship. Similarly, if you have recently shared an affiliation or employment history with the author, it could also be seen to be inappropriate for you to handle their work. Hindawi aims to avoid assigning papers to Editors who might have conflicts, but we also expect our Editors to declare any conflicts. If you believe a conflict exists, you should refuse to handle the manuscript… Reviewers By agreeing to peer review a manuscript you are providing essential neutral assessment. As such, you should ensure that you have no conflicts of interest that could be seen to prevent you from acting in an impartial manner. You should ensure that you have no recent association with the author and that you have not previously coauthored with them. You should also not have a recent shared employment history…”	
IEEE ^[6]^	“Conflict of interest is defined as any situation in which a member’s or volunteer’s decisions or votes could substantially and directly affect the member’s or volunteer’s professional, personal, financial or business interests. …Conflict of Interest Disclosure Statement. IEEE members, non-members or volunteers in an elected or appointed position and volunteers, editors and others involved in making procurement decisions or other activities that could represent a potential conflict of interest as determined by the IEEE Audit Committee shall submit annually a completed Conflict of Interest Disclosure Statement to the Director, IEEE Internal Audit, at the Operations Center. Forms shall be on file within 30 days of assuming his/her position or, in the case of elected positions, within 30 days of acceptance of the nomination, or as otherwise determined by the IEEE Audit Committee. The IEEE staff shall notify every individual requested to file a Conflict of Interest of the applicable deadline. Failure to submit a form shall result in automatic removal from service on the committee, board or election slate, as the case may be.” (IEEE 2017a)	E (R)
	“Individuals involved in making procurement decisions or other activities that could represent a potential conflict of interest must complete the Principles of Business Conduct/Conflict of Interest form every year” (IEEE 2017b)	
Inderscience ^[7]^	As a duty of authors in Author Copyright Agreement.	A
	No exact description on need for declaring COI for editors and reviewers	
NPG ^[8]^	“For the purposes of this statement, competing interests are defined as those of a financial nature that, through their potential influence on behaviour or content or from perception of such potential influences, could undermine the objectivity, integrity or perceived value of a publication…	E, A, R
	Application to authors	
	Unless/until the article is published, authors' declarations will be considered confidential, and will not be disclosed to peer-reviewers.	
	The published article (Article, Letter, Brief Communication, Review, Perspective, Insight) indicates the authors' response using one of the following standard sentences:	
	• The authors declare competing financial interests: details accompany the full-text HTML version of the paper at (url of journal website).	
	• The authors declare no competing financial interests.	
	Application to referees	
	The Nature journals invite peer-reviewers to exclude themselves in cases where there is a significant conflict of interest, financial or otherwise. However, just as financial interests need not invalidate the conclusions of an article, nor do they automatically disqualify an individual from evaluating it. We ask peer-reviewers to inform the editors of any related interests, including financial interests as defined above, that might be perceived as relevant. Editors will consider these statements when weighing reviewers' recommendations.	
	Application to editors	
	All Nature journal editorial staff are required to declare to their employer (Nature Publishing Group) any interests — financial or otherwise — that might influence, or be perceived to influence, their editorial practices. Failure to do so is a disciplinary offence…” (NPG 2017)	
	“Application to authors	
	Authors must disclose and specify any competing interest during the submission process, via declarations in the manuscript submission system. For certain types of content, declarations may be collected via the Nature Research disclosure form. The corresponding author is responsible for providing a declaration on behalf of all authors. For peer reviewed contributions, authors' declarations are disclosed to peer reviewers in full. However, if authors have opted for double-blind peer review, during the peer review process reviewers will be provided with a minimal statement disclosing the existence of any financial or non-financial interest, to prevent the disclosure of authors' identities. Reviewers will be provided the full competing interests declarations at the time of acceptance. Authors opting for double-blind peer review should provide their minimal statement (either "The authors declare the existence of a financial/non-financial competing interest" OR "The authors declare no competing interests") in the submission system and a complete statement of disclosure in their cover letter.	
	In addition to any declarations in submission systems or forms, all authors regardless of peer review model are required to include a statement at the end of their published article to declare whether or not they have any competing interests. The published article indicates the authors' response using one of the following standard sentences:	
	• The authors declare the following competing interests:	
	• The authors declare no competing interests.	
	We recognize that some authors may be bound by confidentiality agreements. In such cases, in place of itemized disclosures, we require authors to state: "The authors declare that they are bound by confidentiality agreements that prevent them from disclosing their competing interests in this work."	
	We do not require authors to state the monetary value of their financial interests.	
	Application to referees	
	The Nature Research journals invite peer-reviewers to exclude themselves in cases where there is a significant conflict of interest, financial or otherwise. However, just as financial interests need not invalidate the conclusions of an article, nor do they automatically disqualify an individual from evaluating it. We ask peer-reviewers to inform the editors of any related interests, including financial interests as defined above, that might be perceived as relevant. Editors will consider these statements when weighing reviewers' recommendations.	
	Application to editors	
	All Nature Research journal editorial staff are required to declare to their employer any interests — financial or otherwise — that might influence, or be perceived to influence, their editorial practices. Failure to do so is a disciplinary offence.” (NPG 2018)	
OUP ^[9]^	“…requires declaration of any Conflict of Interest upon submission. Depending on the journal, you may also be asked to submit signed Conflict of Interest form(s) if your article is accepted for publication…In both cases the corresponding author has to be in a position to report for all co-authors.	E. A. R
	*What is a ‘Conflict of interest’? Any financial interests or connections, direct or indirect, or other situations that might raise the question of bias in the work reported or the conclusions, implications or opinions stated – including pertinent commercial or other sources of funding for the individual author(s) or for the associated department(s) or organization(s), personal relationships, or direct academic competition…*	
	*Who should make the declaration? The corresponding author is expected to obtain the relevant information from all co-authors …*	
	All referees are either asked to decline to review a manuscript if they have a potential conflict or declare any potential conflict.	
	All Editors have submitted a Conflict of Interest statement to the publisher or society. Editors would not handle the review of a manuscript if there was a potential Conflict of Interest, and instead would pass it on to another editorial colleague.”	
PLoS ^[10]^	“Authors, reviewers, and editors must declare potential competing interests, or interests that may be perceived as such, as they relate to the research. A competing interest may relate to a person or an entity and may be of a financial, non-financial, professional or personal nature.”	E, A, R
SAGE ^[11]^	“A Declaration of Conflicting Interests policy refers to a formal policy a journal may have to require a conflict of interest statement or conflict of interest disclosure from a submitting or publishing author…	E, A
	Many scholars, researchers and professionals may have potential conflicts of interest, that could have an effect on – or could be seen to – have an effect on their research. As a result, some SAGE journals require a formal declaration of conflicting interests enabling a statement to be carried within the paginated published article.	
	A potential conflicting interest might arise from relationships, allegiances or hostilities to particular groups, organizations or interests, which may influence excessively one’s judgments or actions. The issue is particularly sensitive when such interests are private and/or may result in personal gain.	
	The same obligations equally apply to editors or guest editors writing an editorial that will be published in the journal.”	
	Also author obligations are defined regarding conflicting interests. “In your Journal Publishing Contributor Agreement you will be asked to certify that:	
	1. All forms of financial support, including pharmaceutical company support, are acknowledged in your Contribution.	
	2. Any commercial or financial involvements that might present an appearance of a conflict of interest related to the Contribution are disclosed in a covering letter accompanying the Contribution and all such potential conflicts of interest will be discussed with the Editor as to whether disclosure of this information with the published Contribution is to be made in the journal.	
	3. That you have not signed an agreement with any sponsor of the research reported in the Contribution that prevents you from publishing both positive and negative results or that forbids you from publishing this research without the prior approval of the sponsor.	
	4 That you have checked the manuscript submission guidelines to see whether the journal requires a Declaration of Conflicting Interests and have complied with the requirements specified where such a policy exists.”	
Springer ^[12]^	“A conflict of interest is a situation in which financial or other personal considerations from authors or reviewers have the potential to compromise or bias professional judgment and objectivity. Authors and reviewers should declare all conflicts of interest relevant to the work under consideration (i.e. relationships, both financial and personal, that might interfere with the interpretation of the work) to avoid the potential for bias.”	A, R
Taylor and Francis ^[13]^	“A conflict of interest can occur when you (or your employer or sponsor) have a financial, commercial, legal, or professional relationship with other organizations, or with the people working with them, that could influence your research.	A
	Full disclosure is required when you submit your paper to a journal. The journal editor will use this information to inform his or her editorial decisions…”	
Wiley ^[14]^	“Editors, authors, and peer reviewers should disclose interests that might appear to affect their ability to present or review work objectively. These might include relevant financial interests (for example, patent ownership, stock ownership, consultancies, or speaker’s fees), or personal, political, or religious interests” and detailed description about tasks, responsibilities of all parties how to avoid and manage COIs.	E, A, R

^[1]^Bentham (2017a) http://benthamscience.com/publishing-ethics-main.php; (2017b) http://benthamscience.com/editorial-policies-main.php; (2017c) http://benthamscience.com/reviewers-guidelines-main.php; (2018) https://benthamscience.com/publishing-ethics-main.php

^[2]^De Gruyter (2017) http://degruyteropen.com/you/journal-author/editorial-policies/other-stm/; (2018) https://www.degruyter.com/view/j/ejnm.2013.5.issue-4/ejnm-2013-0037/ejnm-2013-0037.xml

^[3]^Elsevier (2017a) https://www.journals.elsevier.com/journal-of-accounting-and-economics/policies/conflict-of-interest-policy, Elsevier (2017b) https://www.elsevier.com/conflictsofinterest, Elsevier (2017c) https://www.elsevier.com/about/our-business/policies/publishing-ethics

^[4]^Emerald (2017) http://www.emeraldgrouppublishing.com/authors/writing/best_practice_guide.htm

^[5]^Hindawi (2017) https://about.hindawi.com/managing-conflicts-of-interest/

^[6]^IEEE (2017a) IEEE Policies 2017 https://www.ieee.org/documents/ieee_policies.pdf*; (2017b)*https://www.ieee.org/about/corporate/compliance/business_conduct_and_conflict_of_interest.html

^[7**]**^Inderscience (2017) http://www.icmmcmse2017.org/university/publication/form/Copyright%20Form%20(IJCSE).pdf*(document no longer available)*

^[8]^NPG (Nature Publishing Group) (2017, 2018) http://www.nature.com/authors/policies/competing.html

^[9]^OUP (Oxford University Press) (2017) https://academic.oup.com/journals/pages/authors/authors_faqs/conflicts_of_interest

^[10]^PLoS (2017) https://www.plos.org/editorial-publishing-policies

^[11]^ SAGE (2017 https://uk.sagepub.com/en-gb/eur/declaration-of-conflicting-interests-policy

^[12]^Springer-Nature (2017) https://www.springer.com/gp/authors-editors/editors/publishing-ethics-for-journals/4176#c4230

^[13]^Taylor and Francis / Informa (Routledge) (2017) http://authorservices.taylorandfrancis.com/what-is-a-conflict-of-interest/

^[14]^Wiley (2014) Best Practice Guidelines on Publishing Ethics - Wiley Author Services https://authorservices.wiley.com/asset/photos/Ethics_Guidelines_26.04.17.pdf

Editors and publishers also have the responsibility, as part of a wider process of ensuring accountability and transparency (COPE 2015; Barbour 2017), to expect of themselves what they expect of others. Barbour stated: “If journals want to remain a trusted source of evidence, editors need to step up and apply to themselves the same standards of transparency that they expect of others” (p. 2). We also propose specific responsibilities, some of which were updated in May 2018 in the CSE white paper:

1. explain how editors were recruited and approved and/or show their academic qualifications for that position of responsibility (Teixeira da Silva and Al-Khatib 2018); move away from author-suggested peer reviewers, which may be biased, fake or carry COIs, to an editorial system in which editors are exclusively responsible for conducting peer review themselves or for finding and recruiting suitably qualified peers (Teixeira da Silva and Al-Khatib 2017);
2. ensure that peer review and manuscript processing is kept to a bare minimum, including desk rejections (Teixeira da Silva and Dobránszki 2017);
3. ensure the correction of the literature when errors are detected during PPPR (Teixeira da Silva 2015a);
4. not create accounts for authors without their explicit permission first (Teixeira da Silva 2016c);
5. correct erroneous literature in their journals. Problems associated with literature that was approved by editors, under their supervision and guidance, also forms part of their responsibilities, and thus the professionalism and competence of editors should also be carefully analysed and there must be due process where editors have failed, including their removal from an editorial board (Dobránszki and Teixeira da Silva 2016);
6. ensure the existence of a scientific arbitration board or other formal body that looks at the lack of disclosures between editors and authors or between advertisers and editors to ensure that the choice of selection of publications is not reflecting a hidden commercial or other COIs (Desai and Shortell 2011).

There is concern about editors who have not respected their responsibilities or who may have COIs but who still maintain an editorial position (Teixeira da Silva 2016d). Editorial failure can also include the abuse by editors of their position to either enhance their own work or profiles or to game the metrics system to enhance the metrics of their journal by suggesting excessive citation of their work or of their journal (Teixeira da Silva 2017b), an issue that was also updated in May 2018 in the CSE white-paper. Finally, there is the issue of compensation for editorial duties that carry considerable responsibility. There is continuous debate related to the lack of financial compensation to academics who serve as peer reviewers and editors, especially for the for-profit publishing industry (Teixeira da Silva and Katavić 2016).

## What are COIs in Academia and Publishing?

It is important that the scientific community, including editors, make all possible efforts to ensure the reliability and reproducibility of science through open communication and transparency (Teixeira da Silva and Dobránszki 2018). This is a challenge, especially if editors may be biased by secondary interests, creating a risk in professional judgment or leading to actions regarding a primary interest, a situation that is regarded as a COI (Lo and Field 2009; FCA 2017). A primary interest includes promoting and protecting the integrity and quality of research at all stages while a secondary interest applies to any other issues such as improvement of a financial situation, personal career, or relationships (Lo and Field 2009; FCA 2017).

There are many sources of COIs in the general process of scientific communication (Marcovitch et al. 2010). As Marcovitch et al. (2010) noted:

Reviewers are often unaware that they have potential COI […] —for example, personal friendship (or enmity), religious beliefs or nationalism may get in the way. A reviewer may be over-enthusiastic or unnecessarily hostile about a topic, without realizing he or she is an outlier in that respect. (p. 9)

Paternoster, and Brame (2015), in an editorial, reflect on how an editor or a peer reviewer may become authoritarian. Firstly, very few people decide on whether a paper is accepted or not; secondly, they go beyond their duty to examine the competency of the paper and decide how it should be written; thirdly, editors often abandon their own judgement and base their decisions on the number of reviewers accepting or rejecting the paper; finally, they can and often protect their personal affiliations and values, whether consciously or not (Paternoster and Brame 2015). An authoritarian editor is thus in a position to exercise bias in selecting manuscripts for dissemination. Unlike editorial independence, which is regulated by strict rules, editorial authoritarianism involves personal and professional bias in decision-making.

Depending on the publishing model, the source of the COI can be content-based or inherently financial. Launching a journal, as Momen (2014) argues, requires a large financial outlay, and often subscriptions are not sufficient to meet this initial demand. Financial pressure can lead editors to be conflicted about whether or not to publish an “out of the box” or negative manuscript that challenges mainstream thinking and can adversely affect the inflow of advertisement money. One of the reasons why negative results are not published as often as they should may be because editors display clear content-based bias, cherry-picking positive over negative results—that is, a form of publication bias (Sterling et al. 1995; Teixeira da Silva 2015b)—and thus are serving more as agents selecting what is best for the image of the journal rather than what is best for the good of understanding of the scientific community. Removing results so that editors focus exclusively on methodology in a bid to remove such content- or “positive” results-based bias is also not a realistic solution (Teixeira da Silva 2016e). Within this subset of accepted papers, positive articles may be published before negative ones, which also constitutes publication bias as the speed of publication often impacts the citation and dissemination of the article (Sterling et al. 1995).

Momen (2014) believes that as open access (OA) journals—in contrast to mainstream journals—charge authors, editors are protected from financial pressures and the resultant biases that lead to COIs, as they are not torn between divided loyalties to the journal and its owners on the one hand, and to the author and the scientific community on the other hand. Moreover, and at the same time, they can thus provide space for authors whose research findings do not fit into the mainstream thinking, provided the manuscripts pass peer review and meet the journals’ requirements regarding COIs. However, the author-pays OA model has an inherent financial bias built into its business structure, as the journal can only survive financially when authors (or their institutes) pay article processing fees, as opposed to the traditional subscription-based model, making editorial decisions, to some extent, biased a priori (Al-Khatib and Teixeira da Silva 2017). Of course, this requires that the OA journal have a very strong and clear ethical COI policy for editors. This issue becomes even more pertinent when industry pays editors, as was shown with 64 per cent of 333 “top tier” U.S.-based physician editors from thirty-five journals (Wong et al. 2017). In addition, Paternoster and Brame (2015) argue that the “purpose of a scientific community is to provide a venue for researchers to have a thoughtful discussion about a set of answerable questions” (p. 9). Citing a Paul Krugman, Walter Noll, and Larry Wasserman editorial, Paternoster and Brame (2015) argue that a better way, which is also more democratic and better for science, is to allow the posting of research on the web without any paywalls or passwords so that the entire scientific community has a greater chance to give high-quality comments, including pointing out editorial biases and COIs. What these authors were essentially advocating was the use of open peer review, which discloses the identities of the reviewers, as well as their COIs, although this model has considerable weaknesses and limitations (Teixeira da Silva 2018).

In the editorial process, COIs can be divided into main two groups: financial and non-financial. The most obvious are financial COIs (Janssen et al. 2015) that can be easily identified, for example, business relationships, speaking fees or honoraria for lectures, consulting fees, personal gifts, and others (Cancer Research UK 2014). Both types of COIs can influence professional judgement. Most prestigious journals (80 per cent of journals according to an assessment by Ancker and Flanagin 2007) implemented a COI policy in which researchers (authors) must disclose potential COIs, even if they do not believe that these may influence their study, thereby allowing reviewers and readers to assess if a paper has been affected by any biases. Unfortunately, this policy rarely applies in most journals to reviewers and editors (Cooper et al. 2006). In a survey by Alfonso et al. (2012), among forty-six European cardiovascular journals, it was assessed that 18 per cent of the journals have a specific policy on editors’ COIs. In the same year, Smith et al. (2012) found that about 40 per cent of top-ten peer-reviewed medical journals listed COIs for their editors, but these numbers are unclear for the wider biomedical community journals and even engineering and humanities journals. With respect to editors themselves, in June of 2007, Wager et al. (2009) surveyed 612 EiCs of all medical, healthcare, life science, and social science journals published by Wiley-Blackwell and found that most editors of science journals were neither very concerned about published COIs, nor felt that it was a frequent issue in their journals. Curiously, Wager was the 2009–2012 COPE Chair, while Chris Graf, a Wiley representative and a co-author of that 2009 paper, is the current COPE co-Chair. It is almost self-evident that editors would likely state that indicating their COIs was not important, suggesting that the results of that survey may have been biased or skewed. A more balanced approach would have been to interview editors of journals published by publishers with which Wager and Graf were not associated, and also their authors, that is, the readership, who would most likely perceive editorial COIs more acutely than the editors themselves. Haivas et al. (2014) tested the policies related to editorial COIs of forty peer-reviewed journals on general and internal medicine and found that 60 per cent did not intend to declare their editors’ COIs or those of other advisers in the future. The authors acknowledged their current and past association with the *British Medical Journal* and that the research was funded by the journal and was conducted when the primary and second author were employed by the journal. According to an analysis by Ancker and Flanagin (2007), more than 56 per cent of eighty-four peer-reviewed journals from twelve disciplines had a COI policy specific for editors. Almost 50 per cent of journals published by members of the Japanese Association of Medical Sciences did not require COI disclosures for their editors (Kojima et al. 2015). Verma (2017) concluded that while 67 per cent of eighty-five editorial board members of three American Society for Radiation Oncology journals received some form of payment from industry, either as general payments, research funding, or company ownership, the integrity of peer review was not compromised as a result. Liu et al. (2017) found that only 32.7 per cent of fifty-two “influential (high impact factor for their specialty) US medical journals from 26 specialties” (p. 2) had editor COIs stated clearly, and that just under 51 per cent of eligible editors received some form of payment from pharmaceutical and medical device manufacturers, with 19.5 per cent receiving research payments. Such payments may be subjected to intense and negative media scrutiny and thus cast a negative light on editors and more widely on academia (see for example MacDonald 2018). More recently, fortifying our claims in this paper, Haque et al. (2018) noted that 46 per cent of ten influential U.S. medical journal editors received financial payments but that they had not disclosed these financial COIs, even though they expected their journals’ authors to do so.

Financial COIs are easier to identify and report than non-financial COIs. Janssen et al. (2015) found that 49 per cent of 716 editors of six leading spine journals failed to declare any COIs, but since 42 per cent of editors “with a potential conflict of interest reported a financial relationship of more than $10,000 during the prior year” (6), it was likely that COIs among the 49 per cent group may have been hidden or undeclared, considering that COIs “arising from ties between pharmaceutical industry and physicians are common and may bias research” (2). Mehlman et al. (2017) found that 78 per cent of 908 physician editors of fifteen orthopaedic surgery journals received payments and that the rate of potential editorial COIs ranged from 4 per cent to 73 per cent in the >$10,000 category, from 0 per cent to 31 per cent in the >$250,000 category, and from 0 per cent to 13 per cent in the >$950,000 category. Based on these COIs, the authors concluded that “[a] clear relationship has been established in the literature regarding authors’ potential financial conflicts of interest and study results, and there would seem to be little reason to doubt a similar relationship with respect to journal editors” (p. 4). Only 33 per cent of leading gastroenterology and hepatology journals listed COIs for editors (Qureshi et al. 2012). A PubMed search in 2012 on COIs in medical publishing showed 120 citations between 1991 and 2012, but not one of these citations referred to editorial COIs (Gleicher 2013). These studies leave little doubt about the association between financial COIs and authors or the findings of a study, and it is also quite clear that surprisingly low financial rewards or gifts are able to influence physician behaviour such as prescribing habits or decision-making. Potential financial COIs among editorial boards of the magnitude reported by Mehlman et al. (2017) simply have not been studied. For example, up to 31 per cent of editorial board members of some journals were receiving more than US$250,000/year while only 13 per cent were receiving more than US$950,000/year. Non-financial COIs are more difficult to notice (and judge) by a potentially conflicted or self-conflicted person, making the control of such COIs difficult because they can be highly subjective and because humans are generally not free of COIs (Kozlowski 2016) and as journals usually only require the disclosure of financial COIs. The Agency for Healthcare Research and Quality of the U.S. Department of Health and Human Resources has developed a detailed questionnaire to help identify non-financial COIs, both real and potential, but acknowledges that they cannot be altogether eliminated (Vishwanathan et al. 2013). While journals might not be able to exclude or eliminate editors with such potential financial and non-financial COIs, it would certainly be advantageous to readers for the editorial process to be unblinded at the time of publication to allow readers to make their own independent determination regarding concerns, or the lack thereof, of COIs having influenced the process. It is likely for these reasons that the National Library of Medicine (NLM) and the National Institutes of Health (NIH) implemented on March 8, 2017 the inclusion of a COI statement for all entries on PubMed (Collins 2017), although it is unclear how successfully and how widely this has been implemented and whether all past entries on PubMed would have to retrospectively include a COI statement.

## What do Mainstream Publishers State About Editorial COIs?

Given that academic intellect, owned in part as a result of copyright transfer, is deposited primarily in databases of commercial for-profit publishers, we decided to explore what policies were in place by different publishers, primarily of science, technology, engineering and mathematics, or medicine, regarding editorial COIs (table 1). As indicated in table 1, in 2018, out of the fourteen publishers examined, the COI policy includes a clear rule for requirements of editorial COIs in ten publishers (71 per cent) (De Gruyter, Elsevier, Emerald, Hindawi, IEEE, NPG, OUP, PLOS, SAGE, Wiley) on their websites, but no exact rule was declared in the COI policy of four publishers (Bentham, Inderscience, Springer, and Taylor & Francis/Informa). In several cases, the COI policy applies to authors, editors, and peers, and policies specific to editors cannot be easily teased out. Consequently, to give a global perspective of how these publishers perceive and characterize editorial COIs within the wider context of COIs in their publishing operations, the COI statements verbatim, relating to editors, authors, and peers, have been described in detail in table 1.

A 2013 cross-sectional study that examined 399 JCR (Journal Citation Reports)–indexed biomedical journals showed that almost 90 per cent of these journals required that authors declare financial COIs (just over 70 per cent also required the declaration of non-financial COIs), while the declaration of such COIs by editors was observed in only 38.8 per cent of such journals (Bosch et al. 2013), showing a distinct dichotomy of treatment between authors and editors.

## Displaying Editorial COIs: Suggestions and Advice

To avoid biases emerging from COIs and competing interests and to ensure accountability and transparency through the publishing process, editors’ profiles should carry a simple PDF file next to their names that displays their professional curricula, including publication lists, expertise, and any links to other groups, publishers, professional societies, journals, or networks (Bravo et al. 2017; Herteliu et al. 2017)—that is, full disclosure of information that would allow academics and the public to decide whether such relationships or positions could be construed as actual or possible COIs in their dealings with authors, their functions during peer review, or their responsibilities during PPPR. Networking resulted in 100 per cent more publications by authors who were connected to an editor, which was assessed in 50,000 papers from 30 “major” economics and finance journals (Brogaard et al. 2013). Some examples include by COPE trustees (e.g., the former (2013–2017) COPE Chair, Virginia Barbour), DOAJ editors and management, or even BioMed Central editors, such as the *British Medical Journal* EIC, Fiona Godlee.^2^ In all these cases, a simple PDF file with a list of actual or perceived COIs can be easily incorporated into an editorial board website. The ICMJE provides one such template that editors could use or adapt (ICMJE 2019). Marušić (2009) highlighted the importance of having strict COI-related regulations for editors as such individuals would clearly be most biased towards their own literature or towards publications with which they had personal or professional relationships. Marušić based her definition on the WAME definition of COIs, as was already described above.

The COI forms or statement for editors should include the following information, which should not be limited to one or five years back, as suggested for the JAMA Network journals (Fontanarosa and Bauchner 2017) but in fact reflect a much longer span (at least ten years) of actual or possible COIs:

- A complete and updated educational CV, including academic awards, financial compensation, monetary amounts of grants, names of funding agencies, period of research grants, and links between the grant-awarding body and industry (Teixeira da Silva and Tsigaris 2018). This is because often the grant-awarding body, whether a funding agency or a university, receives finances from the industry for carrying out research. Further, industry representatives may be members of either a board of directors or advisors. For instance, the University of Georgia’s Center for Food Safety offers seats on its board of advisors for $20,000 to industry sponsors, where they can influence and direct the centre’s research efforts. Its advisory board members have included Cargill, ConAgra, General Mills, Unilever, McDonald’s, and Coca-Cola (Food and Water Watch 2012).
- Positions on other editorial boards, indicating the exact editorial position, the journal and publisher name, a URL, start and end period, and remuneration or other benefits as a result of such positions.
- Positions and membership in academic societies and period of membership.
- Links to industry, companies, or any other non-academic organization, voluntary or remunerated (either personally or by a research grant).
- Guest speaker or consultative positions, remuneration, and exact dates of recruitment.

Academia needs to decide if there should be a statute of limitations, for example, listing only five years back, or if none should exist.

Regarding editorial COIs, WAME (2009) states that “(e)ditors should not make any editorial decisions or be involved in the editorial process if they have or a close family member has a COI (financial or otherwise) in a particular manuscript submitted to their journal” (¶3 under “Responsibilities of participants”). For example, if editors have a political or religious COI or a personal COI with respect to the authors or their work, the editors should remove themselves from the decision-making process. Editors may also have a COI if a manuscript is submitted from their own academic department or from their institution; in such situations, they should have explicit policies, made in advance, for how to manage it. When editors submit their own work to their journal, a colleague in the editorial office should manage the manuscript and the editor/author should recuse themselves from discussion and decisions about it (WAME 2009). Although the WAME statement regarding editorial COIs is to be applauded, the issue of how such personal, political, ideological, or religious COIs can be independently verified, if not explicitly stated by the editors, remains a challenge.

The ICMJE requires that editors who make final decisions about manuscripts and guest editors as well as members of the editorial board or advisors should recuse themselves from the decision-making process if COIs, including personal relationships, exist in relation to the article being considered, an opinion also noted by WAME (2009). In addition, editors must regularly publish disclosure statements on potential COIs of journal staff and guest editors (ICMJE 2018).

The guidelines set up to prevent editorial COIs are themselves often inadequate. For instance, COPE’s Code of Conduct for Journal Editors, which is meant to be observed by its members (COPE 2011), advises that in cases of COIs coming to light after publication the journal should publish a correction that discloses the COI, although COPE tend to focus almost exclusively on author-related COIs, deflecting or diluting responsibility associated with editorial COIs. Another COPE document (COPE 2011) related to editorial responsibilities fails to completely acknowledge the existence of editorial COIs. However, a more recent survey has shown that in reality COPE allows its member journals non-compliance with its code and does not exercise its enforcement ability (Ruff 2015). Ruff suggested that “leaders in the scientific community with an impeccable track record of commitment to ethical standards should launch an initiative to set up an independent, effective and credible mechanism, such as a Center for Monitoring and Implementing Publication Ethics” (6). Such a centre, like Amnesty International, would not just create transparency and accountability, but its monitoring and publicly announcement of contraventions would also affect the impact factor of journals (Ruff 2015). One possible example is the New York State Society of CPAs (NYSSCPA).^3^ Currently, there is no independent body to hold COPE, ICMJE, WAME, and other mainstream “ethics” organizations linked to academic publishing (or their members) accountable nor any body to ensure that their actions in academia are transparent.

Further, disclosure of financial COIs itself is not enough to guarantee the prevention of editorial COIs. Prevention and management of COIs requires a broader approach. Editors are public figures and trustees of public good and thus are accountable both to the wider academia and to the public. Issues such as impact factor and biases also affect editorial judgements (Fang and Casadevall 2011). Editors must recognize this and act objectively. The policy in place by PLoS (*PLOS Medicine*^2017^)^4^ for the declaration of non-financial COIs serves as a good starting point. It lists the following as non-financial COIs:

- Acting as an expert witness
- Membership in a government or other advisory board
- Relationship (paid or unpaid) with organizations and funding bodies including non-governmental organizations, research institutions, or charities
- Membership in lobbying or advocacy organizations
- Writing or consulting for an educational company
- Personal relationships (e.g. friend, spouse, family member, current or previous mentor, adversary) with individuals involved in the submission or evaluation of a paper, such as authors, reviewers, editors, or members of the editorial board of a PLOS journal
- Personal convictions (political, religious, ideological, or other) related to a paper’s topic that might interfere with an unbiased publication process (at the stage of authorship, peer review, editorial decision-making, or publication).

Editors must recuse themselves wherever and whenever they feel their judgement may be adversely affected by a non-financial COI. In addition, the following five measures, which reduce the potential of editorial COIs, need to be considered.

First, this paper reveals that not all editors are fully aware of the COIs they bring to their role and that there is need for formal training of academic journal editors in methods of recognition and prevention of all forms of COIs (Wager et al. 2009). A training cell (Ruff 2015) could be established jointly by WAME, COPE, and ICMJE^5^ which would provide training to future and current editors, maybe for a small fee. Journals should be urged to employ such trained editors.

Second, handing over the decision-making process of an article to other editors if it addresses an issue of special interest to the editor. This is being followed by the CMJ and PLOS, is further recommended by the CSE and ICMJE, and is part of Elsevier’s COI policy. The CSE states that:

Editors should avoid making decisions on manuscripts that conflict with their own interest, such as those submitted from their department or by research collaborators, co-authors (in the case of collaborators or co-authors, some time period should be established, such as “for the past five years”), competitors, or those addressing an issue in which they stand to gain financially (e.g., stock in a company whose product is discussed in the article). If they may have a perceived or actual conflict of interest, editors should delegate handling of any decision to other editors with decision-making responsibility. (CSE 2012, ¶3 bullet 1 under “2.1.3 Conflicts of interest”)

According to the ICMJE:

Editors who make final decisions about manuscripts should recuse themselves from editorial decisions if they have conflicts of interest or relationships that pose potential conflicts related to articles under consideration … Editors should publish regular disclosure statements about potential conflicts of interests related to the commitments of journal staff. Guest editors should follow these same procedures. (ICMJE 2018, p. 4)

Elsevier’s COI policy considers relationships between the editor, current and recent colleagues, co-authors, and doctoral students for which the editor served as committee chair as having the potential for COIs. The policy recommends that in such cases, manuscripts should be assigned to co-editors, and if none of the editors satisfy all the conflict screens, the manuscript should be given to the least conflicted co-editor (JAE 2017, table 1).

Third, promotion of OA journals allows a larger readership to exercise control over editorial COIs (Momen 2014). Information and communication technologies have made bulk publishing possible at a low cost which has spawned the birth of several predatory journals, but OA journals, free from financial pressures, can strive to meet the highest standards in publishing (Momen 2014). Today, OA journals which on the whole publish online and do not have any barriers to access, such as paywalls and subscriptions, often have strict COI policies. These journals keep themselves financially afloat by charging authors processing fees, which may differ from a fee of $1,350 per article to publish in *PLoS ONE* to $299 as one-time author fee charged by *PeerJ* to publish an unlimited number of papers by the author. They may also charge membership fees or be subsidized by universities and academic bodies (van Noorden 2013). However, in our opinion, even if content-based bias and COI can be avoided by OA publishing, OA can have an inherent financial bias due to potential risk of an a priori editorial decision (Al-Khatib and Teixeira da Silva 2017), as already described in detail above.

Fourth, in medicine, in order to reduce dissemination bias, clinical trials should be registered, as was recently suggested by the ICMJE, which then backtracked on its promises (Basken 2017), while reporting guidelines should be endorsed. Dissemination bias, which can happen knowingly or unknowingly, includes the editorial decision to delay the publication of the results of a trial because of its negative findings or if there is a competing trial which finishes its manuscript earlier, though the trial itself started later. This can lead to a wrong conclusion that the latter author was the first to form the hypothesis and prove it. Meerpohl et al. (2015) make a strong case for such registration as it helps both in identification of clinical trials as well as the date of initiation of the trial. Thus, it helps to reduce dissemination bias (Meerpohl et al. 2015). Fang and Casadevall (2011) noted that studies with positive results were more likely to be published than studies with negative results which also affected systemic reviews. The authors felt that registration of trials was one measure to ensure that analysis of trials that were reported first are rightly credited and that the credit for the findings are not attributed to trials that have taken place later; this is because registration of trials allows for searching and including relevant studies in systemic reviews (Song et al. 2010). COPE also suggests this in the case of biomedical journals (COPE 2016).

Finally, an ethics committee with credible representative editors and academicians from various disciplines as members, having enforcement ability that allows them to oversee the adherence of journals to the guidelines set up by COPE, WAME, and ICJME, should be established, as was proposed by Ruff (2015). In essence, one or more independent watchdogs are required to monitor the “ethicists” and “ethical organizations” to avoid ethical exceptionalism, that is, a dual system of rules and ethics in which one rule applies to the wider authorship, while a separate and exceptional rule applies exclusively to ethics bodies (Teixeira da Silva 2017c).

## Why Should an Editorial COI Policy Be Applicable to All Journals?

In table 1, we point out that there are no clear policies related to editorial COIs by fourteen mainstream STEMM publishers. Although we recognize that the issue of financial COIs, for example, could be more relevant to biomedical journals where large research funding and corporate interests are often closely linked, some of the larger publishers with journal portfolios that span the hundreds or even thousands of journals would require an editorial COI policy that covers all disciplines, not only biomedicine. This would include theoretical and philosophical journals, including those related to ethics, bioethics, health policy, or scientific integrity. The fact that some journals already have a policy in place to list editorial COIs, or COIs for each editor, suggests that the policy could, and should, be applicable to any, or all, journals, without exception. The objective is to increase transparency and not to suggest that the presence of a bias or COIs by an editor is in some way inherently wrong, simply that it needs to be stated publicly, and openly, in much the same way that authors are expected to make declarations of their own COIs in submitted papers, so that the system is fair and balanced.

## Conclusions

The objective of this paper is to show how accountability and transparency in the publishing process can be increased by expecting editors to display their COIs. There is clear and repeated evidence in the literature and in the rules and guidelines regarding COIs, even among leading publishers (table 1), that excessive and imbalanced emphasis is placed on authors’ COIs and almost none, or very little, on editorial COIs (Resnik and Elmore 2018). Display of editorial COIs should include a detailed account of editors’ professional networks that could be construed as actual or possible influencing factors on several aspects of their editorial duties, such as peer review, processing speed, favouritism towards some authors over others, relationships with other editors or professionals, lobbyists, interest groups, or other ethics- or publishing-related entities, all of which can influence decisions and result in unfair desk rejections or the lack of receptivity to PPPR (Arend 2019), or even perks and favouritism with or among editors or other members of the academic community. This is because editors do not simply represent an independent group of individuals that decide the fate of a submitted paper, but are also the de facto leaders and ambassadors of an academic field. The lack of published editorial COIs may undermine the academic and scholarly objectives of a journal or editorial board to push through viewpoints, perspectives, or policies that have the potential of influencing downstream policies. Or, as stated more eloquently by Leopold et al. (2013, p. 3393), “unmanaged financial COI on an editorial board may influence the development of the scientific literature by affecting what is and is not published.” Academic publishing is becoming increasingly militarized through a flood of measures that are being forcefully implemented to try and curb fraud and recoup trust (Teixeira da Silva 2016f). The issue of bias, including publication bias, which may take on more subtle forms of information manipulation, have not been discussed in this paper and are topics worthy of separate and detailed exploration, even though they may be related to COIs. For example, biases can emerge from COIs or result from the existence of competing interests, financial or otherwise, such as political beliefs or positions (Jena et al. 2018). The Jena et al. paper invoked a strong feedback about the authors’ own COIs (Lomangino 2018). However, it is easy to over-simplify causes and effects, so while COIs may cause biases, do biases cause COIs?

At some point, editors who have largely remained free of scrutiny because academic scrutiny has tended to focus almost exclusively on authors will be increasingly scrutinized as academic publishing attempts to shore up trust and move towards a system that displays editorial COIs publicly, such as on the journal’s website (Thordarson 2017). This is one simple way to ensure trust and fortify accountability and transparency. As one example of setting the right example and following best editorial practices, we praise the initiative by *Anesthesia & Analgesia* to list the COIs of editors.^6^ In contrast, moving forward, journals or publishers that hide editors’ COIs may be perceived as untrustworthy or opaque (Gleicher 2013). In order to achieve true fairness, an editor thus has to give space for manuscripts that do not correspond to, or that challenge, their own ideas as well as contemporary mainstream thinking but which may in future cause a paradigm shift in the understanding of science.

## Footnotes

1
http://www.faseb.org/viewer.aspx?ID=3610&JobPdf=ASB/MBTechnicalImageEditor2017.pdf

2
https://publicationethics.org/files/u661/Virginia%20Barbour%20COIs%20June%202016.pdf; https://doaj.org/about#team; http://www.bmj.com/about-bmj/editorial-staff/fiona-godlee

3
http://www.nysscpa.org/professional-resources/ethics#sthash.iPlAtgYV.dpbs

4 Curiously, one of the *PLOS Medicine* editors when the COI policy was drafted, was Virginia Barbour, emphasizing the notion that rules were created by a small group of individuals that overlapped several organizations (COPE, WAME) and publishers.

5 This advice might be viewed with caution as various members, past and present, within these three organizations are closely linked or have apparent relationships or dealings in editorial boards or ethics associations, so there is a real potential professional COI in this suggestion made by Ruff.

6
https://journals.lww.com/anesthesia-analgesia/Pages/editorialboardprofiles.aspx#jPittet

## References

[CR1] Al-Khatib, A., and J.A. Teixeira da Silva. 2017. Threats to the survival of the author-pays-journal to publish model. *Publishing Research Quarterly* 33(1): 64–70.

[CR2] Alfonso, F. A. Timmis and F.J. Pinto, Editors’ Network European Society of Cardiology Task Force, et al. 2012. Conflict of interest policies and disclosure requirements among European Society of Cardiology National Cardiovascular Journals. *European Heart Journal* 33(5): 587–594. 10.1093/eurheartj/ehr464 22383145

[CR3] Amigo, I., and A. Pascual-García. 2017. Conflicts of interest in scientific publishing. *EMBO Reports* 18(12): 2081–2083. 10.15252/embr.201745008 PMC5709739 29158351

[CR4] Ancker, J.S., and A. Flanagin. 2007. A comparison of conflict of interest policies at peer-reviewed journals in different scientific disciplines. *Science and Engineering Ethics* 13(2): 147–157. 10.1007/s11948-007-9011-z 17717729

[CR5] Arend, R.J. 2019. Conflicts of interest as corrupting the checks and balances in the post-publication oversight of academic business journals. *Journal of Management Inquiry* 28(1): 57–66.

[CR6] Barbour, V. 2017. Competing interests in journal editors. *BMJ* 359: j4819. 10.1136/bmj.j4819 29074639

[CR7] Basken, P. 2017. Journals’ retreat from data-sharing mandate puts onus on universities and government. *The Chronicle of Higher Education*, June 12. http://www.chronicle.com/article/Journals-Retreat-From/240323. Accessed March 7, 2019.

[CR8] Bero, L.A., and Q. Grundy. 2016. Why having a (nonfinancial) interest is not a conflict of interest. *PLoS Biology* 14(12): e2001221. 10.1371/journal.pbio.2001221 PMC5176169 28002462

[CR9] Bik, E.M., F.C. Fang, A.L. Kullas, R.J. Davis, and A. Casadevall. 2018. Analysis and correction of inappropriate image duplication: The *Molecular and Cellular Biology* experience. *Molecular and Cellular Biology* 38(20): e00309-18. 10.1128/MCB.00309-18 PMC6168979 30037982

[CR10] Bosch, X., J.M. Pericas, C. Hernández, and P. Doti. 2013. Financial, nonfinancial and editors’ conflicts of interest in high-impact biomedical journals. *European Journal of Clinical Investigation* 43(7): 660–667. 10.1111/eci.12090 23550719

[CR11] Bravo, G., M. Farjam, F.G. Moreno, A. Birukou, and F. Squazzoni. 2017. Hidden connections: Network effects on editorial decisions in four computer science journals. *Journal of Informetrics* 12(1): 101–112.

[CR12] Brogaard, J., J. Engelberg, and C.A. Parsons. 2013. Networks and productivity: Causal evidence from editor rotations. *Journal of Financial Economics* 111(1): 251–270.

[CR13] Cancer Research UK. 2014. Conflicts of interest policy. http://www.cancerresearchuk.org/funding-for-researchers/applying-for-funding/funding-committees/conflicts-of-interest-guidance-for-committee-members-and-peer-reviewers. Accessed March 7, 2019.

[CR14] CMJ (Croatian Medical Journal). 2009. Guidelines for Authors: Editorial Policy. *Croatian Medical Journal* 50: 93–103.

[CR15] Collins, M. 2017. PubMed updates March 2017. *NLM Technical Bulletin* 415: e2.

[CR16] Committee on Publication Ethics (COPE). 2011. Code of conduct and best practice guidelines for journal editors. https://publicationethics.org/files/Code%20of%20Conduct_2.pdf. Accessed March 7, 2019.

[CR17] COPE. 2011. A short guide to ethical editing for new editors. https://publicationethics.org/files/short%20guide%20to%20ethical%20editing%20for%20new%20editors.pdf. Accessed March 7, 2019.

[CR18] ———. 2015. Principles of transparency and best practice in scholarly writing (version 2). https://publicationethics.org/files/Principles_of_Transparency_and_Best_Practice_in_Scholarly_Publishingv2.pdf. Accessed March 7, 2019.

[CR19] ———. 2016. A short guide to ethical editing for new editors (version 2). https://publicationethics.org/files/A_Short_Guide_to_Ethical_Editing.pdf. Accessed March 7, 2019.

[CR20] Cooper, R.J., M. Gupta, M.S. Wilkes, and J.R. Hoffman. 2006. Conflict of interest disclosure policies and practices in peer-reviewed biomedical journals. *Journal of General and Internal Medicine* 21(12): 1248–1252. 10.1111/j.1525-1497.2006.00598.x PMC1924760 17105524

[CR21] Council of Science Editors. 2019. 2.1 Editor roles and responsibilities [webpage]. https://www.councilscienceeditors.org/resource-library/editorial-policies/white-paper-on-publication-ethics/2-1-editor-roles-and-responsibilities/. Accessed March 7, 2019.

[CR22] Couzin-Frankel, J. 2016. Bringing image manipulation to light. *Science* October 11. 10.1126/science.caredit.a1600143.

[CR23] CSE (Council of Science Editors) 2012. Editors’ roles and responsibilities. CSE White Paper on Publication Ethics. http://cseditors.wpengine.com/resource-library/editorial-policies/white-paper-on-publication-ethics/2-1-editor-roles-and-responsibilities/#213. Accessed March 7, 2019.

[CR24] Desai, S.S., and C.K. Shortell. 2011. Conflicts of interest for medical publishers and editors: Protecting the integrity of scientific scholarship. *Journal of Vascular Surgery* 54(3): 59S–63S. 10.1016/j.jvs.2011.05.111 21872119

[CR25] Dobránszki, J., and J.A. Teixeira da Silva. 2016. Editorial responsibilities: Both sides of the coin. *Journal of Educational and Social Research* 6(3): 9–10.

[CR26] Fang, F.C., and A. Casadevall. 2011. Retracted science and the Retraction Index. *Infection and Immunity* 79(10): 3855–3859. 10.1128/IAI.05661-11 PMC3187237 21825063

[CR27] FCA (Financial Conduct Authority). 2017. FCA Handbook: Systems and Controls. Chapter 10: Conflicts of interest. https://www.handbook.fca.org.uk/handbook/SYSC/10.pdf. Accessed March 7, 2019.

[CR28] Fontanarosa, P., and H. Bauchner. 2017. Conflict of interest and medical journals. *Journal of the American Medical Association* 317(17): 1768–1771. 10.1001/jama.2017.4563 28464125

[CR29] Food & Water Watch. 2012. Public Research, Private Gain – Corporate Influence over University Agricultural Research – A Report. https://www.foodandwaterwatch.org/sites/default/files/Public%20Research%20Private%20Gain%20Report%20April%202012.pdf. Accessed March 7, 2019.

[CR30] Funk, C. 2017. Real numbers: Mixed messages about public trust in science. *Issues in Science and Technology* 34(1). http://issues.org/34-1/real-numbers-mixed-messages-about-public-trust-in-science/. Accessed March 7, 2019.

[CR31] Gallo, S.A., M. Lemaster, and S.R. Glisson. 2016. Frequency and type of conflicts of interest in the peer review of basic biomedical research funding applications: self-reporting versus manual detection. *Science and Engineering Ethics* 22(1): 189–197. 10.1007/s11948-015-9631-7 25649072

[CR32] Gleicher, N. 2013. Avoiding currently unavoidable conflicts of interest in medical publishing by transparent peer review. *Reproductive BioMedicine Online* 26(5): 411– 415. 10.1016/j.rbmo.2013.01.015 23507135

[CR33] Gottlieb, J.D., and N.M. Bressler. 2017. How should journals handle the conflict of interest of their editors? Who watches the “watchers”? *Journal of the American Medical Association* 317(17): 1757–1758. 10.1001/jama.2017.2207 28464152

[CR34] Haivas, I., S. Schroter, F. Waechter, and R. Smith. 2014. Editors’ declaration of their own conflicts of interest. *Canadian Medical Association Journal* 171(5): 475–476. 10.1503/cmaj.1031982 PMC514645 15337729

[CR35] Haque, W., A. Minhajuddin, A. Gupta, and D. Agrawal. 2018. Conflicts of interest of editors of medical journals. *PLoS ONE* 13(5): e0197141. 10.1371/journal.pone.0197141 PMC5959187 29775468

[CR36] Herteliu, C., M. Ausloos, B.V. Ileanu, G. Rotundo, and T. Andrei. 2017. Quantitative and qualitative analysis of editor behavior through potentially coercive citations. *Publications* 5(2): 15.

[CR37] ICMJE (International Committee of Medical Journal Editors). 2018. Recommendations for the conduct, reporting, editing, and publication of scholarly work in medical journals. http://www.icmje.org/icmje-recommendations.pdf. Accessed March 7, 2019. 10.12771/emj.2024.e48 PMC12093628 40704003

[CR38] ———. 2019. Conflicts of interest [webpage]. http://icmje.org/conflicts-of-interest/. Accessed March 7, 2019.

[CR39] *JAE* (*Journal of Accounting and Economics*) 2017. Conflict of interest policy. https://www.journals.elsevier.com/journal-of-accounting-and-economics/policies/conflict-of-interest-policy. Accessed March 7, 2019.

[CR40] Janssen, S.J., A.L. Bredenoord, W. Dhert, M. de Kleuver, F.C. Oner, and J.J. Verlaan. 2015. Potential conflicts of interest of editorial board members from five leading spine journals. *PLoS ONE* 10(6): e0127362. 10.1371/journal.pone.0127362 PMC4456083 26042410

[CR41] Jena, A.B., A.R. Olenski, A. Khullar, A. Bonica, and H. Rosenthal. 2018. Physicians’ political preferences and the delivery of end of life care in the United States: retrospective observational study. *BMJ* 361: k1161. 10.1136/bmj.k1161 PMC5894480 29643089

[CR42] *Journal of Medical Imaging and Radiation Sciences*. 2013. Conflict of interest policy. http://www.jmirs.org/pb/assets/raw/Health%20Advance/journals/jmir/Editorial_Board_Conflict_Policy.pdf. Accessed March 7, 2019.

[CR43] Kesselheim, A.S., J.L. Lee, J. Avorn, A Servi, W.H. Shrank, and N.K. Choudry. 2012. Conflict of Interest in oncology publications: a survey of disclosure policies and statements. *Cancer* 118(1): 188–195. 10.1002/cncr.26237 21717432

[CR44] Kojima, T., J. Green, and J.P. Barron. 2015. Conflict-of-interest disclosure at medical journals in Japan: A nationwide survey of the practices of journal secretariats. *BMJ Open* 5: e007957. 10.1136/bmjopen-2015-007957 PMC4554913 26310399

[CR45] Kozlowski, L.T. 2016. Coping with the conflict-of-interest pandemic by listening to and doubting everyone, including yourself. *Science and Engineering Ethics* 22(2): 591–596. 10.1007/s11948-015-9658-9 PMC4819729 26025653

[CR46] Kullas, A.L., and R.J. Davis. 2017. Setting the (scientific) record straight: *Molecular and Cellular Biology* responds to post-publication review. *Molecular and Cellular Biology* 37(11): e00199-17. 10.1128/MCB.00199-17 PMC5440648 28512216

[CR47] Lenzer, J. 2016. When is a point of view a conflict of interest? *BMJ* 355: i6194. 10.1136/bmj.i6194 27881367

[CR48] Leopold, S.S L. Beadling, L.M.B. Dobbs, et al. 2013. Active management of financial conflicts of interest on the editorial board of CORR. *Clinical Orthopaedics and Related Research* 471: 3393–3394. 10.1007/s11999-013-3279-x PMC3792242 24057190

[CR49] Liu, J.J., C.M. Bell., J.J. Matelski, A.S. Detsky, and P. Cram. 2017. Payments by US pharmaceutical and medical device manufacturers to US medical journal editors: Retrospective observational study. *BMJ* 359: j4619. 10.1136/bmj.j4619 PMC5655612 29074628

[CR50] Lo, B. and M.J. Field. eds. 2009. *Conflict of Interest in Medical Research, Education, and Practice*. Institute of Medicine (U.S.) Committee on Conflict of Interest in Medical Research, Education, and Practice, Washington DC: National Academies Press (U.S.). https://www.ncbi.nlm.nih.gov/pubmed/20662118. Accessed March 7, 2019. 20662118

[CR51] Lomangino, K. 2018. Uncovering new peer review problems—The time at the BMJ. *Health News Review*, April 18. https://www.healthnewsreview.org/2018/04/uncovering-new-peer-review-problems-this-time-at-the-bmj/. Accessed March 7, 2019.

[CR52] MacDonald, F. 2018. This is the sickening amount pharmaceutical companies pay top journal editors. *ScienceAlert*, April 12. https://www.sciencealert.com/how-much-top-journal-editors-get-paid-by-big-pharma-corrupt. Accessed March 7, 2019.

[CR53] Marcovitch, H., V. Barbour, C. Borrell, et al. 2010. Conflict of interest in science communication: More than a financial issue: Report from Esteve Foundation Discussion Group, April 2009. *Croatian Medical Journal* 51(1): 7–15. 10.3325/cmj.2010.51.7 PMC2829174 20162740

[CR54] Marušić, A. 2009. Editorial interest in conflict of interest. *Croatian Medical Journal* 50(4): 339–341. 10.3325/cmj.2009.50.339 PMC2728390 19673032

[CR55] McCoy M.S., and E.J. Emanuel. 2017. Why there are no “potential” conflicts of interest. *Journal of the American Medical Association* 317(17): 1721–1722. 10.1001/jama.2017.2308 28464154

[CR56] Meerpohl, J.J., L.K. Schell, D. Bassler, et al. 2015. Evidence-informed recommendations to reduce dissemination bias in clinical research: Conclusions from the OPEN (Overcome failure to Publish nEgative fiNdings) project based on an international consensus meeting. *BMJ Open* 5: e006666. 10.1136/bmjopen-2014-006666 PMC4431130 25943371

[CR57] Mehlman, C.T., K. Okike, , M. Bhandari, and M.S. Kocher. 2017. Potential financial conflict of interest among physician editorial board members of orthopaedic surgery journals. *Journal of Bone and Joint Surgery* 99(5): e19. 10.2106/JBJS.16.00227 28244918

[CR58] Momen, H. 2014. Institutional journals as an alternative model for open access. *Memórias do Instituto Oswaldo Cruz* 109(7): 847–848. 10.1590/0074-0276140334 PMC4296487 25410986

[CR59] Paternoster, R. and R. Brame. 2015. Isn’t it time to consider alternatives to traditional peer review? *The Criminologist* 40(6): 9–10.

[CR60] *PLOS Medicine* Editors. 2008. Making sense of non-financial competing interests. *PLOS Medicine* 5(9): e199. 10.1371/journal.pmed.0050199 PMC2553825 18828670

[CR61] *PLOS Medicine* 2017. Competing interests. http://journals.plos.org/plosmedicine/s/competing-interests. Accessed March 7, 2019.

[CR62] PLOS One. 2019. Competing interests [webpage]. http://journals.plos.org/plosone/s/competing-interests. Accessed March 7, 2019.

[CR63] Qureshi, J., A. Sud, and N. Vakil. 2012. Funding source and conflict of interest disclosures by authors and editors in gastroenterology specialty journals revisited. *Alimentary Pharmacology and Therapeutics* 35(6): 690–695. 10.1111/j.1365-2036.2011.04989.x 22257079

[CR64] Resnik, D.B., and S.A. Elmore. 2018. Conflict of interest in journal peer review. *Toxicologic Pathology* 46(2): 112–114. 10.1177/0192623318754792 PMC5825276 29382273

[CR65] Ruff, K. 2015. Scientific journals and conflict of interest disclosure: What progress has been made? *Environmental Health* 14(1): 45. 10.1186/s12940-015-0035-6 PMC4450857 26024916

[CR66] Rumsey, T.S. 1999. One editor’s views on conflict of interest. *Journal of Animal Science* 77(9): 23792383. 10.2527/1999.7792379x 10492443

[CR67] Sarigöl, E., D. Garcia, I. Scholtes, and F. Schweitzer. 2017. Quantifying the effect of editor–author relations on manuscript handling times. *Scientometrics* 113(1): 609–631. 10.1007/s11192-017-2309-y PMC5629258 29056793

[CR68] Smith, E., M.J. Potvin, and B. Williams-Jones. 2012. Accessibility and transparency of editor conflicts of interest policy instruments in medical journals. *Journal of Medical Ethics* 38(11): 679–684. 10.1136/medethics-2012-100524 22556312

[CR69] Smith, N.L. 2002. An analysis of ethical challenges in evaluation. *The American Journal of Evaluation* 23(2): 199–206.

[CR70] Song, F., S. Parekh, L. Hooper, et al. 2010. Dissemination and publication of research findings: An updated review of related biases. *Health Technology Assessment* 14(8): iii, ix-xi, 1–193. 10.3310/hta14080 20181324

[CR71] Sterling, T.D., W.L. Rosenbaum, and J.J. Weinkam. 1995. Publication decisions revisited—The effect of the outcome of statistical tests on the decision to publish and vice versa. *The American Statistician* 49(1): 108112.

[CR72] Teixeira da Silva, J.A. 2013. Responsibilities and rights of authors, peer reviewers, editors and publishers: A status quo inquiry and assessment. *The Asian and Australasian Journal of Plant Science and Biotechnology* 7(Special Issue 1): 6–15.

[CR73] ———. 2015a. A call for greater editorial responsibilities. *Science Editing* 2(2): 89–91.

[CR74] ———. 2015b. Negative results: negative perceptions limit their potential for increasing reproducibility. *Journal of Negative Results in BioMedicine* 14(1): 12. 10.1186/s12952-015-0033-9 PMC4494691 26149259

[CR75] ———. 2016a. Ethical center-piece for genetics and genomics studies: Broad advice on figure and gel manipulation. *Biomedical Genetics and Genomics* 1(1): 3–4.

[CR76] ———. 2016b. Retractions represent failure. *Journal of Educational and Social Research* 6(3): 11–12.

[CR77] ———. 2016c. On the abuse of online submission systems, fake peer reviews and editor-created accounts. *Persona y Bioética* 20(2): 151–158.

[CR78] ———. 2016d. Do zombie scientists and editors exhibit hubris, or blind courage? *Focus on Sciences* 2(4): 2.

[CR79] ———. 2016e. Does the removal of results from a submitted paper reduce publication bias? *Pacific Science Review B: Humanities and Social Sciences* 2(1): 2930.

[CR80] ———. 2016f. The militarization of science, and subsequent criminalization of scientists. *Journal of Interdisciplinary Medicine* 1(2): 214–215.

[CR81] ———. 2017a. Fake peer reviews, fake identities, fake accounts, fake data: Beware! *AME Medical Journal* 2: 28.

[CR82] ———. 2017b. The ethics of peer and editorial requests for self-citation of their work and journal. *Medical Journal Armed Forces India* 73(2): 181–183. 10.1016/j.mjafi.2016.11.008 PMC5592272 28924321

[CR83] ———. 2017c. Ethical exceptionalism: Can publishing rules be manipulated to give the impression of ethical publishing? *Bangladesh Journal of Medical Science* 16(4): 610–614.

[CR84] ———. 2018. Challenges to open peer review. *Online Information Review* (in press) 10.1108/OIR-04-2018-0139.

[CR85] Teixeira da Silva, J.A., and A. Al-Khatib 2017. How are editors selected, recruited and approved? *Science and Engineering Ethics* 23(6): 1801–1804. 10.1007/s11948-016-9821-y 27896601

[CR86] ———. 2018. Should authors be requested to suggest peer reviewers? *Science and Engineering Ethics* 24(1): 275–285. 10.1007/s11948-016-9842-6 28155093

[CR87] Teixeira da Silva, J.A., and J. Dobránszki. 2015. Problems with traditional science publishing and finding a wider niche for post-publication peer review. *Accountability in Research: Policies and Quality Assurance* 22(1): 22–40. 10.1080/08989621.2014.899909 25275622

[CR88] ———. 2017. Excessively long editorial decisions and excessively long publication times by journals: Causes, risks, consequences, and proposed solutions. *Publishing Research Quarterly* 33(1): 101–108.

[CR89] ———. 2018. Editors moving forward: Stick to academic basics, maximize transparency and respect, and enforce the rules. *Recenti Progressi in Medicina* 109(5): 263–266. 10.1701/2902.29244 29771248

[CR90] Teixeira da Silva, J.A., and V. Katavić. 2016. Free editors and peers: Squeezing the lemon dry. *Ethics & Bioethics* 6(3–4): 203–209.

[CR91] Teixeira da Silva, J.A., and P. Tsigaris. 2018. Academics must list all publications on their CV. *KOME* 6(1): 94–99.

[CR92] Thordarson, D.B. 2017. Conflict of interest and FAI. *Foot & Ankle International* 38(5): 471. 10.1177/1071100717705363 28457166

[CR93] van Noorden, R. 2013. Open access: The true cost of science publishing. *Nature* 495(7442): 426–429. 10.1038/495426a 23538808

[CR94] Verma, V. 2017. Financial relationships with industry of editorial board members of the three journals of the American Society for Radiation Oncology. *International Journal of Radiation Oncology and Biological Physics* 99(2): 286–291. 10.1016/j.ijrobp.2017.03.020 28871971

[CR95] Vishwanathan, M., T.S. Carey, S.E. Belinson, E. Berliner, S. Chang et al. 2013. Identifying and Managing Nonfinancial Conflicts of Interest for Systematic Reviews. Agency for Healthcare Research and Quality. Available at https://www.ncbi.nlm.nih.gov/books/NBK148586/. Accessed March 7, 2019. 23844449

[CR96] Wager, E., S. Fiack, C. Graf, A. Robinson, and I. Rowlands. 2009. Science journal editors’ views on publication ethics: Results of an international survey. *Journal of Medical Ethics* 35(6): 348–353. 10.1136/jme.2008.028324 19482976

[CR97] WAME (World Association of Medical Editors). 2009. Conflict of interest in peer-reviewed medical journals. http://wame.org/conflict-of-interest-in-peer-reviewed-medical-journals. Accessed March 7, 2019.

[CR98] Wicherts, J.M. 2017. The weak spots in contemporary science (and how to fix them). *Animals* 7: 90. 10.3390/ani7120090 PMC5742784 29186879

[CR99] Wiersma, M., I. Kerridge, and W. Lipworth. 2018. Should we try to manage non-financial interests? *BMJ* 361: K1240. 10.1136/bmj.k1240 29650517

[CR100] Wong, V.S.S., L.N. Avalos, and M.L. Callaham. 2017. Industry payments to physician journal editors. *PLoS One* 14(2): e0211495. 10.1371/journal.pone.0211495 PMC6366761 30730904

